# Reporting of adverse drug reactions: an exploratory study among nurses in a teaching hospital, Ajman, United Arab Emirates

**DOI:** 10.1186/2008-2231-20-44

**Published:** 2012-10-04

**Authors:** Lisha Jenny John, Mohamed Arifulla, Jenny John Cheriathu, Jayadevan Sreedharan

**Affiliations:** 1 Lecturer, Department of Pharmacology, Gulf Medical University, Ajman, United Arab Emirates; 2 Professor and Head, Department of Pharmacology, Gulf Medical University, Ajman, United Arab Emirates; 3 Lecturer and Specialist, Department of Pediatrics, Gulf Medical College Hospital and Research Center, Ajman, United Arab Emirates; 4 Professor and Assistant Director, Research Division, Gulf Medical University, Ajman, United Arab Emirates

## Abstract

**Background and the purpose of the study:**

Adverse drug reactions (ADRs) are important public health problem associated with morbidity, mortality and financial burden on the society. Nurses play important role in medication safety surveillance through the spontaneous voluntary reporting of ADRs. Nurses’ knowledge, attitude and practice towards ADR reporting and factors affecting reporting was assessed in the study.

**Methods:**

All nurses working in a tertiary care hospital, Ajman, UAE participated in this cross-sectional survey. A self administered questionnaire of four domains (knowledge, attitude, practice, factors affecting reporting) was distributed among nurses after obtaining informed consent. The knowledge and attitude components were assigned score of one for correct response. Data was analyzed using SPSS (version 19). Mann–Whitney *U* test was used to compare knowledge and attitude scores between subgroups; Spearman’s correlation for any relationship between knowledge and attitude.

**Results:**

Of the total participants, females constituted 92.3%; average duration of clinical experience 6.5 ± 3.3 years; mean age 28.9 ± 4.1 years. Median score for knowledge components of ADR reporting was 11(total score: 17) and for attitude components was 4(total score: 8). No difference noted in knowledge and attitude scores between gender, age group, educational qualification. A positive correlation between knowledge and attitude components was observed (r = 0.38). ADRs are important cause for morbidity and mortality was reported by (54.9%). 49.5% were aware of Pharmacovigilance centers’. Uncertainty of ADRs (49.5%); concern that the report may be wrong (46.2%) and inadequate knowledge of ADR reporting procedure were the major barriers to reporting. Training in ADR reporting as the key measure to improve reporting was suggested by (86.8%).

**Major conclusion:**

The results of the study strongly point out the need for interventional program among nurses focusing on the importance of ADR reporting and reporting procedure to encourage their active, voluntary participation in drug safety surveillance.

## Introduction

Adverse drug reactions (ADRs) are one of the major drug related problems associated with pharmacotherapy. ADRs are important public health problem imposing a considerable economic burden on the society and health-care systems. It is one of the important causes of hospitalization varying between 5-13% [1-4]. Spontaneous and voluntary reporting system is an integral component of drug safety surveillance program and also the most effective methods of acquiring ADR information especially the new and serious ADRs. The spontaneous reporting system of the pharmacovigilance program has contributed significantly to improve the ADR reporting rates worldwide [5,6]. Nevertheless, under-reporting is the major shortcoming of spontaneous reporting system [7,8]. Under-reporting delays early detection of ADRs and can increase associated morbidity, mortality in the patient. Health care professionals play an important role in the detection, assessment and spontaneous reporting of ADRs. Several studies have established the role of health care providers in spontaneous reporting of ADRs [9-11].

Among the health care providers, nurses are in a unique position to monitor and report ADRs. Knowledge and attitude of nurses towards reporting of ADRs play a significant role in the spontaneous reporting system. Several factors influence reporting behaviour among health care providers such as; financial incentives for reporting; fear of litigation; belief that serious ADRs are well documented; uncertainty of an ADR, a single ADR report may not contribute and lack of interest or lack of time [12]. Identifying the factors influencing reporting is essential to suggest measures to enhance reporting. Several studies carried out to assess the knowledge, attitude, and practice among nurses have documented that the knowledge of ADR reporting procedure are inadequate among nurses [11,13-17]. There have been no empirical studies from United Arab Emirates evaluating the knowledge, attitude, and practice of ADR reporting among nurses. Hence the present study aimed to assess the knowledge, attitude, and practice of nurses to ADR reporting and factors that influence reporting in a multispecialty teaching hospital.

## Methods

### Study design and setting

Nurses working at Gulf Medical College Hospital and Research Center (GMCHRC), Ajman, UAE participated in this cross-sectional survey. GMCHRC is the first private multi-specialty teaching hospital in the Emirate of Ajman.

### Study population

During the study period (March 2011- May 2011) there were a total of 110 nurses working in the hospital and they were approached to participate in the study. The nurses who were not willing to participate in the study and those on leave during the study period were excluded.

### Study tool

The knowledge, attitude, and practice of ADR reporting among the nurses were assessed by using a self administered questionnaire which included both open-ended and closed-ended questions. It included the following domains:

1. Demographic information of participants.

2. Seventeen questions about the knowledge of nurses regarding adverse drug reaction, pharmacovigilance, importance of ADR reporting, location of ADR monitoring center, and ADR reporting system.

3. Eight questions related to the attitude of nurses towards pharmacovigilance.

4. Practice of reporting of ADRs. The questions on practice in the questionnaire included whether they have reported any ADR, number of times they have reported ADR to Pharmacovigilance center.

5. Factors encouraging and discouraging reporting of ADRs.

Knowledge questions mainly centered on general concept of pharmacovigilance and adverse drug reaction reporting system. Attitude questions focused on the nurses’ viewpoint regarding different aspects of ADR reporting. The nurses response ranged from strongly agree, strongly disagree and not sure for the attitude questions. A score of one was assigned to the correct response to questions. Thus the maximum possible score for knowledge component was 17 and eight for attitude questions.

### Data collection

The approval from the Institutional Ethics Committee was sought before the initiation of the study. The questionnaires were distributed to nurses in all the hospital wards and outpatient departments after obtaining their informed consent. The anonymously filled in questionnaires were collected back on the same day.

### Data management and statistical analysis

The collected data were entered into the Microsoft Excel spread sheet, and analyzed using SPSS statistical software (version 19). Mann–Whitney *U* test was used for comparing the median scores for knowledge and attitude components among different subgroups of respondents such as gender, age group variation and educational qualification. Spearman’s correlation was used to determine any relationship between knowledge and attitude. All tests were carried out at significance level of less than or equal to 0.05.

## Results

### Demographic characteristics

A total of 91 nurses (response rate of 82.7%) were included in the study. The vast majority of respondents 84 (92.3%) were females, with males representing 7.7% of the total; this reflects the gender imbalance within health care services especially in the nursing field. Considering years of experience in the field of nursing, the experience ranged from one year to eighteen years with average clinical experience of 6.5 ± 3.3 years. Respondents’ ages ranged from 20 to 45 years, with a mean of 28.9 ± 4.1 years. Table 1 shows the demographic distribution of the participants.

**Table 1:** Demographic characteristics of the participants

**Item**	**Sub group**	**Number**	**Percentage**
Age group			
	< 30 years	55	60.4
	> = 30 years	36	39.6
Years of experience			
	</=5 years	39	42.9
	>5 years	52	57.1
Qualification			
	BSc Degree	21	23.1
	Diploma	70	76.9
Gender			
	Male	7	7.7
	Female	84	92.3
Nationality			
	Pakistani	2	2.2
	Indian	84	92.3
	Egyptian	1	1.1
	Filipino	4	4.4

### Knowledge and attitude scores

The total possible score for knowledge component was 17. The median score for knowledge on ADR reporting was 11 and the scores ranged from five to 16. In about 27 nurses (29.7%), the scores varied from 11 to 13 (65-76% of the total knowledge score); and 26 (28.5%) nurse’s the scores varied from 14 to 16 (82-94% of the total knowledge score). The correct definition of pharmacovigilance and ADR was identified by 76(83.5%) of the nurses. Of the total, only 50(54.9%) nurses knew that ADRs are important cause for morbidity and mortality and 24(26.4%) responded that they were not aware of this fact.

The total possible score for attitude components was eight; the median score for attitude components was four and the minimum score was zero and maximum eight. A total of 37(40.7%) nurses stated ADR reporting is a professional obligation.

The comparison of median scores for the knowledge and attitude with demographic variables is shown in Table 2. Nurses less than 30 years of age had a slightly higher knowledge scores than those above 30 years of age which was not statistically significant. Similarly the nurses with BSc degree had slightly higher median knowledge score than the Diploma holders. The median attitude score was similar in both age groups as well as level of education. The knowledge and attitude scores of nurses with previous experience in reporting ADRs and those not reported, were similar in the two groups; implying that their previous experience of reporting did not improve their knowledge or attitude towards reporting (Table 2).

**Table 2:** Demographic characteristics and the knowledge, attitude scores

**Item**	**Subgroup**	**Knowledge component**	**p value**	**Attitude component**	**p value**
**Age**					
	< 30 years	12		4	
	>/=30 years	10.5	0.23	4	0.95
**Nursing experience**					
	<=5 years	12		4	
	>5 years	11	0.07	4	0.63
**Qualification**					
	B.Sc degree	12		4	
	Diploma degree	11	0.16	4	0.47
**Gender**					
	Male	12		3	
	Female	11	0.51	4	0.18
**Previous experience in ADR reporting**					
	Yes	10		3.5	
	No	12	0.23	4	0.58

The Spearman correlation was applied for knowledge and attitude components and the co-efficient obtained was 0.38 which suggests a positive correlation; as the knowledge of ADR reporting increases among the nurses, their attitude towards reporting also improves accordingly.

### Practice component

A total of 85(82.4%) nurses had observed ADRs in their nursing practice. Even though ADR reporting is not mandatory, eight (8.8%) nurses had reported ADRs to the Pharmacovigilance centers. All the nurses had reported the ADRs to the concerned doctor as soon as they noticed. 45(49.5%) respondents were aware of the existence of Pharmacovigilance centers.

### Factors encouraging ADR reporting

The factors that promote reporting of ADRs stated by the participants were; patient safety 80(87.9%), the seriousness of the ADR 69(75%) and new ADRs 69(75%).

### Barriers to ADR reporting

The factors that hinder reporting among the nurses were; uncertainty of the ADRs 45(49.5%); concern that the report may be wrong 42(46.2%) and inadequate knowledge of the ADR reporting procedure 41(45%). The other factors that discourage nurses from reporting ADRs are listed in Table 3.

**Table 3:** Barriers to reporting of ADRs by the nurses

**Barriers to reporting**	**Number**	**Percentage**
Uncertainty of the ADRs	45	49.5
Report may be wrong	42	46.2
Unaware of the reporting procedure	41	45.1
Lack of time to fill-in a report	30	33.0
Need not report a recognized ADR	29	31.9
Reporting may generate extra work	24	26.4

**Figure 1:**
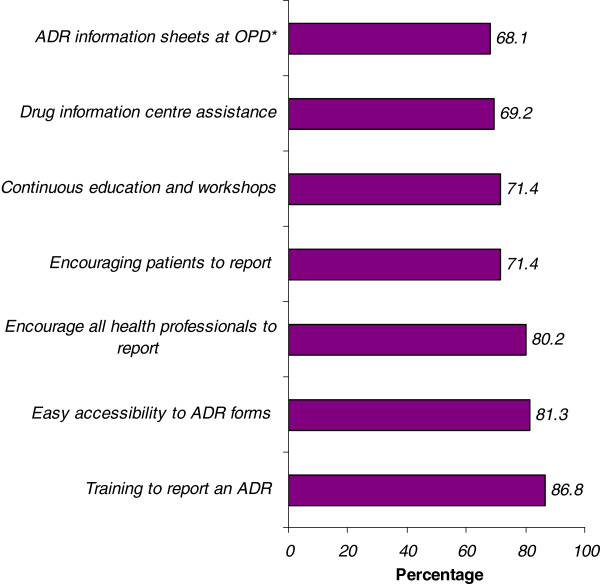
**Strategic approaches suggested by the respondents to enhance ADR reporting.** * OPD: Out Patient Department; ADR: Adverse drug reaction.

The most common strategic approaches suggested by the respondents to enhance the reporting frequency were; training to report ADRs and easy accessibility to ADR reporting forms. The other measures suggested by the participants have been depicted in the Figure 1.

Only five of the nurses had received training in the reporting process, but majority of the nurses were willing to be trained 79 (86.8%).

## Discussion

Health care providers play an integral role in the Pharmacovigilance program especially the nurses due to their close interaction with the patients. Nurses are often the primary source of possible ADR alerts to the clinicians.

Majority of the respondents in the study were females (92.3%). This finding was similar to previous studies [16,17]. Generally it is observed that the females opt for nursing career than the males which explains this finding on gender disparity. Majority of the nurses in our study were Diploma holders in Nursing, unlike Hanafi et al. where in, majority were Graduates (BSc degree) [16]. The average age of nurses in our study ranged between 20-45 years, which was similar to the earlier studies among the nurses [16,17].

In the present study, about half of the respondents were aware that ADRs are an important cause for morbidity and mortality. In contrast, Fadare et al. noted that 93.8% of their study participants were aware of this fact regarding ADRs [18].

The knowledge scores for more than 50 percent of the nurses were above 65% of the total score. The attitude scores of ADR reporting for about 28% of the participants were above 50%. One of the important finding in the present study was the positive correlation between knowledge and attitudes towards ADR reporting. Thus if the knowledge on ADR reporting is improved then the nurses’ attitude also improves which would be reflected on the ADR reporting schemes in a positive manner. In distinction, Hanafi et al. reported poor knowledge level among the nurses and positive attitude to towards Pharmacovigilance [16]. Palaian et al. noted low scores for both knowledge and attitude components of ADR reporting among the nurses [15].

The correct definition of the terms ‘pharmacovigilance’ and ‘adverse drug reaction’ was identified by 76(83.5%) of the nurses, similar to an earlier study from Iran [16]. In contradiction, Rajesh R et al. and Hanafi et al. studies reported that 21.3% and 32% of the nurses (pre intervention) identified the correct definition of pharmacovigilance respectively [14,17] and only 1.6% of nurses in a study from China [13].

Hajebi G et al. reported that nurses with prior familiarity to ADR center had greater knowledge and positive attitude to towards ADR reporting [17]. However in the present study comparing the scores of nurses who have had previously reported ADR and those not reported, the knowledge and attitude scores were similar suggesting that the previous experience of reporting did not improve their knowledge or attitude towards reporting. The probable reason for this finding could be the lack of continuum in the reporting process; ADR reporting is a continuous process among the health care providers in the hospital.

Considerable number of the nurses (80%) had observed ADRs during their nursing practice, and all of them had reported to the concerned doctors and 8.8% of the nurses had reported ADRs to Pharmacovigilance center in spite of ADR reporting not being mandatory similar to other reports [13,17,19,20]. This shows the nurses concern towards their patients' safety. In contradiction, Fadare et al. noted that about 74% of the nurses had observed ADRs [18]. Soleymani et al., demonstrated the significant role of spontaneous reporting system in the health care system [21]. Soleymani et al. reported that the overall reporting frequency of tramadol induced ADRs reduced drastically over a period of 5 years (2006-2010) which was attributed to the initiatives taken by the National Pharmacovigilance center. High reporting frequency of tramadol induced severe ADRs and poisoning cases (dose dependent) to the Iranian Pharmacovigilance Center by spontaneous reporting lead to the implementation of new guidelines to limit the distribution of tramadol to hospital use only and also changed the potency of available injectable form from100mg to 50 mg [21]. These examples can be presented to understand importance of spontaneous reporting in patient safety.

According to the results, 50% of the participants were aware of the existence of ADR monitoring centers. This finding was comparable to Khalili et al. from Iran [20]. This observation further emphasizes the need for awareness of the ADR reporting system among the nurses.

About 40% of the nurses in the present study felt ADR reporting is a professional obligation. Similar observation was noted by a previous report from Iran (16). Patient safety is the prime responsibility of the nurses and by the active and voluntary participation in the Pharmacovigilance program they contribute towards their patient’s safety and medical ethics.

The major barriers to under-reporting, from the present study were uncertainty of the ADRs, the concern of the report may be wrong and inadequate knowledge of the reporting procedure. These observations were consistent with the earlier reports [13,18,20]. These observations reflect the common anxieties among the reporters. An ADR report need not be confirmatory of the relationship between the drug and ADR. When an ADR is suspected, even those not known to the drug it should be reported. In order to address the problem of under-reporting, ADR reporting procedure can be made available as small booklets, posters, electronic flashes at various locations in the hospital to serve as constant reminders. Regular sensitization programs such as continued nursing education and workshops should be carried out among nurses to stress the importance of pharmacovigilance. These training programs clear all misconceptions associated with the ADR reporting.

The strategic approaches suggested by the respondents to enhance reporting included training in ADR reporting and easy accessibility to ADR reporting forms which is in line with Li et al. Training and educational interventions would increase the knowledge and as knowledge is directly related to the attitude, this would in turn motivate nurses to reports the ADRs they encounter. Previous reports have reported significant improvement of knowledge, attitude and perception of healthcare workers about ADR after intervention [20,22,23] Educational program can include presentations, workshops, small group discussions, providing information about pharmacovigilance for healthcare workers by mail, newsletters, reminders, advertisement and continuous education of nurses, and involvement of clinical pharmacists in the medical wards [20]. Pharmacovigilance can be included in the nursing curriculum to introduce them to this concept early in their career. Nursing supervisors and administrators can monitor and actively initiate these program and workshops to improve reporting in the hospitals. Regular feed back of the reports and presentations of the reported ADRs can be a motivational force to continue reporting process among the health care professionals.

The limitations of the study include; the results are of a single-centre and small sample size which may be. The study can be further extended to other hospitals in the country to generalize the findings. Another limitation is those inherent to questionnaire-based studies such as subjective response and recall bias.

In conclusion, the results of the study strongly point out the need for interventional program among nurses focusing on the importance of ADR reporting and reporting procedure to encourage their active, voluntary participation in drug safety surveillance.

## Competing interests

The authors declare that they have no competing interests.

## Authors’ contributions

LJ designed the study, deduced the data, drafted the manuscript, and revised it. JC acquired the data. LJ, JA, MA planned the study, conducted the data analysis, interpreted the data, and revised the manuscript. JS participated in statistical analysis, interpreted the data, and revised the manuscript. MA, JC, JS critically revised the manuscript. All the authors approved the final document.
